# Supplementary material for: Content-rich biological network constructed by mining PubMed abstracts
Source: BMC Bioinformatics. 2004 Oct 8;5:147. doi: 10.1186/1471-2105-5-147 (PMC528731; doi:10.1186/1471-2105-5-147)
Supplement: Additional File 2 — The original results of the above study (non-essential files are deleted to keep the file size under the limit set by BMC bioinformatics). [file 1471-2105-5-147-S2.bz2 › chilibotAdditionalFile2/dip10/397ID8663535E9951/html/EGFR_AREG.html]

 


 **EGFR** and **AREG** 
  
Found 112 abstracts in PubMed, retrieved 10.  
 

 What does Google say? 
 PDF only 
| .edu only 

---

**Interactive relationship** (e.g. stimulation, inhibition, etc)

**Neutral relationship**- ... the activation of  **EGFR**  in response to smoke involves cleavage of amphiregulin  [ **AREG** ]  by ADAM 17.  Ref: 12568494 Novartis Found Symp, 2002

**Non-interactive relationship** (e.g. studied together, co-existance, homology, etc.)

- Similarly,  **EGFR**  TK inhibition by ZD1839 reversed the invasive phenotype promoted by the constitutively activated form of the  **EGFR**  EGFRvIII and the  **EGFR**  agonists transforming growth factor alpha TGFalpha, amphiregulin  [ **AREG** ]  and EGF.  Ref: 12881705 Oncogene, 2003
- The binding of amphiregulin  [ **AREG** ]  to  **EGFR**  then stimulates proliferation of lung epithelial cells.  Ref: 12711607 J Biol Chem< MedlineTA>J Biol Chem, 2003
- RT PCR transcripts of  **EGFR**  ligands EGF, TGF alpha, HB EGF, and amphiregulin  [ **AREG** ]  were detected in both carcinomas and normal mucosa, indicating that autocrine growth stimulation of colorectal carcinomas is mediated by coexpression of EGF receptor ligands and upregulation of EGF receptors.  Ref: 12790326 Recent Results Cancer Res, 2003
- Our results point to the involvement of several ErbB  [ **EGFR** ]  specific ligands amphiregulin  [ **AREG** ]  and neuregulin 1 and enzymes or adaptor molecules PI3K, Src, Shc and Grb7 in the ErbB  [ **EGFR** ]  pathway dysregulation associated with breast cancer.  Ref: 12866037 Int J Cancer, 2003
- The smoke triggered mechanism includes the generation of oxygen radicals, which in turn stimulate tumor necrosis factor alpha converting enzyme TACE, ADAM 17 metalloproteinase to cleave transmembrane amphiregulin  [ **AREG** ] , a ligand for the epidermal growth factor receptor  [ **EGFR** ]   **EGFR** .  Ref: 12711607 J Biol Chem< MedlineTA>J Biol Chem, 2003
